# A Large Language Model-Based Approach for Coding Information from Free-Text Reported in Fall Risk Surveillance Systems: New Opportunities for In-Hospital Risk Management

**DOI:** 10.3390/jcm14051580

**Published:** 2025-02-26

**Authors:** Davide Rango, Giulia Lorenzoni, Henrique Salmazo Da Silva, Vicente Paulo Alves, Dario Gregori

**Affiliations:** 1 Unit of Biostatistics, Epidemiology and Public Health, Department of Cardiac, Thoracic, Vascular Sciences and Public Health, University of Padova, 35131 Padova, Italy; davide.rango@ubep.unipd.it (D.R.); dario.gregori@unipd.it (D.G.); 2 Pós-Graduação em Gerontologia, Universidade Catolica de Brasilia, Brasília 71966-700, DF, Brazil; henrique.salmazo@p.ucb.br (H.S.D.S.); vicerap@gmail.com (V.P.A.)

**Keywords:** large language models, risk management, in-hospital falls, free text

## Abstract

**Background/Objectives:** Falls are the most common adverse in-hospital event, resulting in a considerable social and economic burden on individuals, their families, and the healthcare system. This study aims to develop and implement an automatic coding system using large language models (LLMs) to extract and categorize free-text information (including the location of the fall and any resulting injury) from in-hospital fall records. **Methods**: The study used the narrative description of the falls reported through the Incident Reporting system to the Risk Management Service of an Italian Local Health Authority in Italy (name not disclosed as per research agreement). The OpenAI application programming interface (API) was used to access the generative pre-trained transformers (GPT) models, extract data from the narrative description of the falls, and perform the classification task. The GPT-4-turbo models were used for the classification task. Two independent reviewers manually coded the information, representing the gold standard for the classification task. Sensitivity, specificity, and accuracy were calculated to evaluate the performance of the task. **Results**: The analysis included 187 fall records with free-text event descriptions detailing the location of the fall and 93 records providing information about the presence or absence of an injury. GPT-4-turbo showed excellent performance, with specificity, sensitivity, and accuracy values of at least 0.913 for detecting the location and 0.953 for detecting the injury. **Conclusions**: The GPT models effectively extracted and categorized the information, even though the text was not optimized for GPT-based analysis. This shows their potential for the use of LLMs in clinical risk management research.

## 1. Introduction

Falls are the most common adverse in-hospital event, with yearly cases in U.S. hospitals ranging from 700,000 to 1 million (roughly 2 falls per 100 hospitalized patients) [1]. About one-fourth of falls result in some injury [1], potentially leading to loss of independence and increased length of stay [2]. Due to their consequences, fall-related adverse events impose a considerable social and economic burden on individuals, their families, and the healthcare system [2].

The Global Patient Safety Action Plan recognizes in-hospital falls as avoidable harms and sets their prevention as a priority to improve patient safety [3]. A key strategy to mitigate the impact of falls is to assess each patient’s fall risk upon admission. This requires a thorough evaluation of their fall history, including occurrence patterns, circumstances, contributing factors, and physical, cognitive, psychological, and social conditions [4]. Categorizing patients based on their risk of falling can facilitate the implementation of tailored primary prevention measures, helping to reduce the occurrence of falls during hospitalization [5].

Countries worldwide have put forward several risk management programs to reduce the burden of in-hospital injuries. To this end, in Italy, the Ministry of Health has issued Recommendation No. 13, “Prevention and management of patient falls in healthcare facilities”, which applies to all healthcare environments and professionals. This initiative aims to improve patient safety and implement a data collection system for adverse in-hospital events, including fatal falls and those causing severe injuries. In such a way, a systematic collection of these events has been established to guarantee epidemiological surveillance of the phenomenon [6].

Despite these interventions, the problem of falls in healthcare facilities remains relevant. A potential explanation might be the insufficient implementation of risk management interventions within hospital environments. Furthermore, it has been shown that there is a substantial lack of consistency about specific topic areas, such as the usefulness of vitamin D supplementation for fall prevention [7]. In this context, continuing the epidemiological surveillance of the phenomenon is essential to contribute to its understanding.

However, in several countries, the in-hospital fall data collection system is not standardized, making epidemiological surveillance challenging. Artificial intelligence may help improve the epidemiological surveillance of the phenomenon by exploiting free-text information often reported in medical records, which has been shown to substantially benefit patients [8].

The advent of large language models (LLMs) has the potential to introduce a paradigm shift in clinical research [9,10]. The literature has suggested the potential for the use of LLMs within biomedical data, e.g., for data extraction and classification, and the preliminary results are promising [11]. These models can address various tasks, including clinical and research-related activities [12]. The use of LLMs to extract information from health records has already been documented in injury surveillance, showing promising results. In particular, a study published in 2024 used a large sample of unstructured medical notes to identify fall-related injuries with excellent performance [13]. A recent systematic review also investigated the use of natural language processing for fall prediction and detection in healthcare settings, identifying 26 different studies [14]. Furthermore, in a broader context, other studies have employed LLMs to extract injury information from emergency department records involving adult and pediatric patients [15,16].

This work is focused on developing and implementing an automated coding system using LLMs to extract and categorize free-text information (including the location of the fall and any resulting injury) from in-hospital fall records reported to the risk management system of an Italian Local Health Authority, assessing the feasibility of such models in clinical risk management.

## 2. Materials and Methods

This analysis examined in-hospital falls reported through the incident reporting system to the Risk Management Service of a Local Health Authority in Italy (name not disclosed as per research agreement). This Local Health Authority includes two hospitals and three health districts. A similar analysis on a different subset of the data was previously published [17]. Ethical review and approval were not required since the study was retrospective in nature and used anonymized data.

The data collection form had two parts. The first included information about the patient and the characteristics of falls. The second collected information on the outcomes of the fall. All information was collected in a structured manner, except for a narrative description of the event, which was requested at the end of the data collection form.

The present analysis focused on the narrative description of the fall. For this reason, only the subset of records reporting a narrative description specifying the fall location and if an injury occurred were included in the analysis.

### 2.1. Reference Method: Manual Classification

Information on each fall’s location and the associated injuries was retrieved from free-text descriptions. These descriptions were then manually classified into specific categories by two experienced clinicians:

(1) location of the fall: hospital bathroom, hospital room, hallway;
(2) fall-related injuries (any physical harm or damage to the body resulting from the fall, including impacts such as hitting the head or any other body part): yes vs. no.

The two clinicians were blinded to the classification process. A third, independent reviewer resolved any disagreements. All records were anonymous and carefully reviewed to ensure no risk of data disclosure. The text was written in Italian.

### 2.2. GPT-Based Classification

The LLM used for this task was GPT-4 Turbo, a version of the generative pre-trained transformer (GPT) model. The OpenAI application programming interface (API) was used to access the model, extract data from the narrative descriptions of the falls, and perform the classification task. The openai R package [18] was used to interface with the GPT-4 Turbo models, facilitating remote computations without local computational loads.

Different parameter configurations were used for “temperature” and “frequency and presence penalty”. Temperature controls the randomness of model responses. A higher temperature increases the randomness, leading to more varied and sometimes less predictable results. The “frequency and presence penalty” parameters influence text output by reducing the probability of word repetition. The frequency component of the penalty decreases the likelihood of repeating words that have appeared multiple times in the text, promoting lexical diversity. In contrast, the presence component penalizes the repetition of any word after its initial use, regardless of its frequency. Higher positive values reinforce the penalties against word repetition.

The temperature and frequency/presence penalty were tested for different combinations of values from 0.2 to 1.2 to determine the optimal settings for extracting the specific, deterministic information suitable for direct use in statistical analysis.

Prompts were generated according to OpenAI recommendations [19] and are presented in Table 1. They were developed through an iterative process in which an initial version was tested and refined several times based on model responses until the desired quality and clarity were achieved.

### 2.3. Statistical Analysis

Descriptive statistics were reported as percentages (absolute numbers) for the categorical variables. GPT’s performance was evaluated by calculating the accuracy, sensitivity, and specificity, along with bootstrap 95% confidence intervals (CI) within 1000 repetitions. All analyses were performed using R software version 4.3.2 and the openai libraries.

## 3. Results

The database comprised 187 fall records containing free-text descriptions of the events that specify the fall location (Table 2) and 93 records that indicate whether an injury occurred (Table S1 Supplementary Material).

According to the gold-standard criterion, among the 187 records detailing the location, 67% (126 cases) occurred in the hospital bedroom, while 30% (56 cases) occurred in the bathroom. Only five cases occurred in the hospital hallways (Table 2).

Regarding the 93 cases detailing the presence or absence of fall-related injury in the free-text event description, 64 records reported injury (Table S1, Supplementary Material).

Table 3 presents the performance metrics for location detection (no calculations were performed for the hallway, since only five cases were reported, and no misclassification occurred). GPT-4-Turbo showed excellent performance, with specificity, sensitivity, and accuracy values of at least 0.913. The combination of parameters that provided the best accuracy was 0.7 for both the temperature and the frequency/presence penalty. For this value, the accuracy of correctly identifying falls occurring in the hospital room was 0.947 (95% CI 0.914, 0.973). The sensitivity was 0.944 (95% CI 0.900, 0.978), and the specificity was 0.951 (95% CI 0.886, 1.000). Similar performance values were obtained also for bathroom falls. Table S2 (Supplementary Material) presents the classification agreement for each one of the parameter combinations (temperature and frequency/presence penalty) of GPT-4-Turbo. No classification errors were detected for cases in the hallway for all parameter combinations.

Table 4 presents the performance metrics for the detection of injuries. The performance was excellent, with sensitivity, specificity, and accuracy values of at least 0.953. The combination of 1.2 temperature and 1.2 frequency/presence provided perfect specificity, high accuracy, 0.978 (95% CI 0.946, 1.000), and sensitivity, 0.969 (95% CI 0.919, 1.000). Table S3 (Supplementary Material) presents the classification agreement.

Table S4 presents an example of a misclassified record. The case was complex because, even if not explicitly stated, it can be inferred that the fall occurred in the hospital room since the patient was found near the bed. Probably, the fact that it explicitly mentioned the bathroom was the main reason why the record was wrongly classified.

## 4. Discussion

This study aimed to demonstrate LLMs’ feasibility and added value in clinical risk management research, focusing on in-hospital fall surveillance. Furthermore, it compared different combinations of temperature and presence-penalty parameters. The performance was impressive, and no substantial variability in the GPT performance was detected at different temperatures or frequency/presence penalty levels. This work represents a re-analysis (of a different subset of data) of a previously published paper [17]. In the previous work, structured data were integrated with free-text descriptions of the falls, employing topic modeling to identify significant themes that emerged from the narratives. While exploiting the unstructured text, that method focused primarily on discovering higher-level patterns and categories. In the present study, however, free-text narratives were exclusively analyzed and exploited using an LLM to automatically extract two specific pieces of information: the location of each fall event and whether or not it caused an injury. As a result, the method takes full advantage of the richness of free-text reports, which are often underutilized in many contexts, providing a more complete picture of the circumstances and outcomes of falls, which can significantly improve prevention and management efforts.

From the clinical perspective, the study aims to supplement—rather than replace—physicians’ manual coding by extracting key information that would otherwise remain unused because it is exclusively contained in free-text narratives.

The present results are promising. They highlight the potential for LLMs to be valuable in non-English-speaking settings, emphasizing their capability to process multilingual healthcare data effectively. Implementing automated systems capable of analyzing and categorizing complex information from risk management data collection systems creates opportunities for a new paradigm in clinical risk management research [20]. Interestingly, the results are consistent with those presented in the literature, showing the high performance of LLMs, including LLMs other than GPT, in data extraction and classification [13].

A detailed and automatic analysis of the causes and consequences of in-hospital falls would provide essential aid for supporting epidemiological surveillance of the phenomenon. Extracting information effectively from extensive collections of unstructured data could enable early risk detection, the development of tailored intervention protocols, and predictive analysis to enhance patient safety in hospital settings.

The use of LLMs in biomedical data is still in the early stages. The literature shows demonstrative applications of natural language processing tasks in the biomedical field, with generally impressive results. For example, LLMs have demonstrated superior performance to physicians in clinical text synthesis [11]. They have also shown excellent performance in extracting information from medical notes, with GPT models showing remarkably better performance than other LLMs [21].

Despite the promising results, it is noteworthy that the use of LLMs in clinical research is often debated, especially regarding their ethical implications. Ethical considerations are critical when using LLMs to analyze clinical patient data, particularly regarding privacy, data protection, and fairness. As the World Health Organization highlighted, safeguarding patient confidentiality requires strong governance structures and careful oversight of data collection, storage, and use [22]. Data security measures, such as encryption or in-house processing, help prevent unauthorized access and promote trust between patients and healthcare providers. Equity is also critical. Models trained on unrepresentative or biased datasets risk reproducing bias, exacerbating inequities in health care delivery. In addition, questions about accountability and transparency persist when LLMs provide clinical recommendations that physicians and patients cannot fully interpret. Despite these challenges, responsible and ethically grounded implementation of LLMs in medicine has the potential to improve diagnostic accuracy, streamline administrative processes, and optimize resource allocation, ultimately improving patient outcomes. As research progresses and models perform better, LLMs can be increasingly integrated into standard clinical practice, provided their development and use are subject to strict ethical and regulatory frameworks [23].

### 4.1. Study Limitations

A primary limitation of this study was the small dataset, particularly concerning injury outcomes. Only 93 contained information on the existence of an injury, and of these, only 64 indicated the presence of an injury without standardized details on severity. Given these constraints, our analysis was necessarily limited to a binary classification (lesion present or absent), and the model’s performance should not be over-interpreted for more complex tasks, such as stratifying lesion severity or identifying specific mechanisms of injury. Assessing the reliability of an LLM-based classification model in such a limited dataset is challenging, as small sample sizes can lead to overestimating performance metrics and reducing the generalizability of results.

A related limitation was the single-center nature of the study, which further limits the representativeness of the dataset and may introduce selection bias. Differences in reporting practices, hospital protocols, and patient populations could influence how falls are documented, affecting the performance of any automated classification approach. However, this problem stems from the study design rather than the methodological framework itself. LLMs for automatic text classification are scalable tools that can be applied to larger, multi-institutional datasets, allowing for a more comprehensive assessment of their robustness in different healthcare settings. Expanding the dataset in future studies will be essential to confirm the broader applicability of this approach.

Another study limitation should also be noted. The fact that the model used for the extraction and classification tasks operates as a “black box” makes it difficult to understand why it classifies some records correctly and fails to classify others accurately. This is one of the main reasons why the scientific community views LLMs in biomedical research with skepticism [24]. However, concerning the typical “black-box” nature of LLMs, which significantly impacts interpretability, methods from the field of explainable artificial intelligence (such as local interpretable model-agnostic explanations—LIMEs—or Shapley additive explanations—SHAPs) could, in principle, be adapted to textual analyses by modifying or occluding portions of text to observe how these changes affect the output, thus identifying which semantic components most influence GPT classification. Although applying these methods to LLMs remains an evolving area of research, it could provide greater transparency and build confidence in the clinical adoption of these tools. Anyway, we must recognize that functioning as a black box is the main drawback of all artificial intelligence algorithms [25], including those developed in-house. This problem fuels the ongoing debate about the challenges and potential of using artificial intelligence in medical research. Perhaps in the future, we will accept artificial intelligence models as valuable resources for clinical and research activities, accepting that “the ability to explain how results are produced can be less important than the ability to produce such results and empirically verify their accuracy” [26].

Finally, the study employed only one LLM (GPT). Future work could evaluate the performance of multiple language models (e.g., bidirectional encoder representations from transformers—BERT) to evaluate the differences in accuracy, interpretability, and computational requirements. These direct comparisons could better clarify whether a particular architecture is better suited for similar tasks. Anyway, the GPT model was chosen primarily because it is currently one of the most widely used language models in the medical literature, facilitating comparison with the results from other studies.

### 4.2. Clinical Implications

Unstructured free-text descriptions remain a suboptimal but often unavoidable component of hospital safety documentation. Although structured fields exist, they are often incomplete or fail to capture the full context of an event. In this scenario, LLM-based methodologies represent a promising approach to extract, categorize, and standardize clinically relevant information, improving the usability of risk management data. This study demonstrates how automated extraction of fall location and injury details can support surveillance efforts by providing a scalable tool for hospital safety monitoring and risk assessment.

From a clinical perspective, improving the processing of fall reports has direct implications for patient safety and prevention strategies. Systematic extraction of fall location data can help hospitals identify high-risk areas, enabling targeted environmental modifications and tailored fall prevention programs. In addition, integrating the injury data extracted from the LLM with clinical risk factors (such as frailty, motor disability, or polypharmacy) could enable more sophisticated predictive models for fall risk stratification. This knowledge could guide clinicians in developing personalized prevention measures, reducing the incidence of in-hospital falls and the associated complications.

In addition to supporting prevention strategies, automatic text classification improves the quality and consistency of risk reporting. By aligning narrative descriptions with coded data, LLMs can help detect inconsistencies, standardize terminology, and ensure that critical details are not underestimated. This process can serve as a quality control mechanism, enabling hospital administrators and risk managers to refine documentation workflows and improve the accuracy of adverse event reporting. In addition, the ability to process large volumes of free-text incident reports enables both retrospective and real-time monitoring, making safety interventions more timely and data-driven.

Although this study focused on a relatively small dataset from a single institution, the potential for broader implementation is significant. Future research should evaluate the integration of LLM-based tools into electronic health records and clinical decision support systems, assessing their role in real-time surveillance and automated risk stratification. In addition, multicenter validation is needed to determine the generalizability of this approach in different healthcare settings.

## 5. Conclusions

This study investigated the potential of GPT-based models for the automated extraction and analysis of fall data in hospital settings. This approach allows free-text reports to be systematically analyzed, reducing the need for manual review and ensuring that relevant information is identified and used correctly. Improving the accessibility and organization of fall data can help hospitals monitor falls more efficiently and support targeted prevention strategies. Finally, a significant added value of the study should be pointed out. Although the text was not explicitly optimized for GPT-based processing, the model showed excellent performance in effectively extracting and categorizing information, highlighting the LLMs’ potential to be used with routine clinical practice data.

## Figures and Tables

**Table 1:** GPT prompts for extraction of relevant information.

Completion Sequence	GPT Prompt Text
**System**	“This is a dataframe containing records of accidental falls in healthcare facilities. Each record corresponds to a fall that may have occurred in a specific location within the hospital”.
**System**	“In the first column is reported the id of the accidental fall. In the second column is reported the description of the fall”.
**System**	“The text is in Italian, however, responses must be provided strictly in English using the specified category terms”.
**User**	Location “Where did the fall occur? You must choose only one of the following options: ‘hospital bathroom’, ‘hospital room’, ‘hallway’. Use only the words corresponding to these categories and use only lowercase. Do not write any other output or comments. Do not deviate from the provided options”. Fall-related physical harm “Classify any event where the patient sustained physical harm, such as injuries or noticeable impacts, regardless of severity. Examples include hitting a body part or any other physical trauma. Did the patient sustain any injuries, either immediately or as a result of the fall? You must choose only one of the following options: ‘Yes’, ‘No’. Use only the words corresponding to these categories and use only lowercase. Do not write any other output or comments. Do not deviate from the provided options”.

**Table 2:** Characteristics of the fall records with free-text event descriptions detailing the location. Data are absolute numbers (percentages).

Characteristic	N = 187
Witnesses of the fall	
No	91 (49%)
Yes	95 (51%)
Fall risk management plan available	
No	34 (19%)
Yes	142 (81%)
Previous in-hospital falls (same hospitalization)	
No	164 (90%)
Yes	18 (10%)
Potential causes (patient)	
Walking barefoot	42 (34%)
Open slippers	45 (37%)
Walking aids	7 (6%)
Type of clothing	4 (3%)
Medical devices (e.g., drainages)	6 (5%)
More than one	19 (15%)
Location (gold standard)	
Bathroom	56 (30%)
Hospital bedroom	126 (67%)
Hallway	5 (3%)

**Table 3:** Performance metrics according to the location. For each metric, point estimates (95% confidence interval) are reported for different combinations of temperature and frequency/presence penalty.

	Hospital Room	Bathroom
Temperature: 0.2, Frequency/Presence penalty: 0.2		
Accuracy	0.925 (0.888, 0.963)	0.925 (0.888, 0.963)
Sensitivity	0.913 (0.861, 0.961)	0.946 (0.885, 1.000)
Specificity	0.951 (0.894, 1.000)	0.916 (0.867, 0.962)
Temperature: 0.2, Frequency/Presence penalty: 0.7		
Accuracy	0.925 (0.888, 0.963)	0.925 (0.888, 0.963)
Sensitivity	0.913 (0.862, 0.960)	0.946 (0.883, 1.000)
Specificity	0.951 (0.895, 1.000)	0.916 (0.866, 0.962)
Temperature: 0.2, Frequency/Presence penalty: 1.2		
Accuracy	0.925 (0.882, 0.957)	0.925 (0.882, 0.957)
Sensitivity	0.913 (0.857, 0.957)	0.946 (0.877, 1.000)
Specificity	0.951 (0.885, 1.000)	0.916 (0.862, 0.959)
Temperature: 0.7, Frequency/Presence penalty: 0.2		
Accuracy	0.925 (0.888, 0.963)	0.925 (0.889, 0.963)
Sensitivity	0.913 (0.862, 0.959)	0.946 (0.879, 1.000)
Specificity	0.951 (0.887, 1.000)	0.916 (0.867, 0.960)
Temperature: 0.7, Frequency/Presence penalty: 0.7		
Accuracy	0.947 (0.914, 0.973)	0.947 (0.914, 0.973)
Sensitivity	0.944 (0.900, 0.978)	0.946 (0.877, 1.000)
Specificity	0.951 (0.886, 1.000)	0.947 (0.903, 0.978)
Temperature: 0.7, Frequency/Presence penalty: 1.2		
Accuracy	0.931 (0.893, 0.963)	0.931 (0.893, 0.963)
Sensitivity	0.921 (0.869, 0.966)	0.946 (0.887, 1.000)
Specificity	0.951 (0.894, 1.000)	0.925 (0.874, 0.967)
Temperature: 1.2, Frequency/Presence penalty: 0.2		
Accuracy	0.931 (0.893, 0.963)	0.931 (0.893, 0.963)
Sensitivity	0.913 (0.862, 0.961)	0.964 (0.912, 1.000)
Specificity	0.967 (0.919, 1.000)	0.916 (0.867, 0.962)
Temperature: 1.2, Frequency/Presence penalty: 0.7		
Accuracy	0.931 (0.893, 0.963)	0.931 (0.893, 0.963)
Sensitivity	0.913 (0.861, 0.961)	0.964 (0.908, 1.000)
Specificity	0.967 (0.915, 1.000)	0.916 (0.866, 0.962)
Temperature: 1.2, Frequency/Presence penalty: 1.2		
Accuracy	0.931 (0.888, 0.963)	0.931 (0.888, 0.963)
Sensitivity	0.921 (0.865, 0.963)	0.946 (0.878, 1.000)
Specificity	0.951 (0.889, 1.000)	0.924 (0.872, 0.964)

**Table 4:** Performance metrics for fall-related injuries. For each metric, point estimates (95% confidence interval) are reported for different combinations of temperature and frequency/presence penalty.

Temperature: 0.2, Frequency/Presence penalty: 0.2	
Accuracy	0.968 (0.925, 1.000)
Sensitivity	0.969 (0.918, 1.000)
Specificity	0.966 (0.889, 1.000)
Temperature: 0.2, Frequency/Presence penalty: 0.7	
Accuracy	0.957 (0.914, 0.989)
Sensitivity	0.953 (0.894, 1.000)
Specificity	0.966 (0.893, 1.000)
Temperature: 0.2, Frequency/Presence penalty: 1.2	
Accuracy	0.968 (0.925, 1.000)
Sensitivity	0.969 (0.919, 1.000)
Specificity	0.966 (0.880, 1.000)
Temperature: 0.7, Frequency/Presence penalty: 0.2	
Accuracy	0.968 (0.935, 1.000)
Sensitivity	0.969 (0.921, 1.000)
Specificity	0.969 (0.921, 1.000)
Temperature: 0.7, Frequency/Presence penalty: 0.7	
Accuracy	0.968 (0.925, 1.000)
Sensitivity	0.969 (0.921, 1.000)
Specificity	0.966 (0.893, 1.000)
Temperature: 0.7, Frequency/Presence penalty: 1.2	
Accuracy	0.978 (0.946, 1.000)
Sensitivity	0.984 (0.950, 1.000)
Specificity	0.966 (0.885, 1.000)
Temperature: 1.2, Frequency/Presence penalty: 0.2	
Accuracy	0.968 (0.925, 1.000)
Sensitivity	0.969 (0.919, 1.000)
Specificity	0.966 (0.889, 1.000)
Temperature: 1.2, Frequency/Presence penalty: 0.7	
Accuracy	0.968 (0.925, 1.000)
Sensitivity	0.969 (0.921, 1.000)
Specificity	0.966 (0.885, 1.000)
Temperature: 1.2, Frequency/Presence penalty: 1.2	
Accuracy	0.978 (0.946, 1.000)
Sensitivity	0.969 (0.919, 1.000)
Specificity	1.000 (1.000, 1.000)

## Data Availability

The raw data supporting the conclusions of this article will be made available by the authors upon request.

## Supplementary Materials

The following supporting information can be downloaded at https://www.mdpi.com/article/10.3390/jcm14051580/s1: Table S1. Characteristics of the fall records with free-text event descriptions detailing the presence or absence of fall-related injuries. Data are absolute numbers (percentages). Table S2. Confusion matrix by location. The columns report the classification according to the gold standard. The rows present the classification according to GPT for all possible combinations of the values of temperature and frequency/presence penalty. Data are absolute numbers (percentages). Table S3. Confusion matrix for fall-related injury. The columns report the classification according to the gold standard. The rows present the classification according to GPT for all possible combinations of the values of temperature and frequency/presence penalty. Data are absolute numbers (percentages). Table S4. Example of record misclassified (fall in the hospital bedroom misclassified as fall in the bathroom).

## References

[1] LeLaurin J.H., Shorr R.I. (2019). Preventing Falls in Hospitalized Patients: State of the Science. Clin. Geriatr. Med..

[2] Morello R.T., Barker A.L., Watts J.J., Haines T., Zavarsek S.S., Hill K.D., Brand C., Sherrington C., Wolfe R., Bohensky M.A. (2015). The Extra Resource Burden of In-Hospital Falls: A Cost of Falls Study. Med. J. Aust..

[3] World Health Organization (2021). Global Patient Safety Action Plan 2021–2030: Towards Eliminating Avoidable Harm in Health Care.

[4] Appeadu M.K., Bordoni B. (2023). Falls and Fall Prevention in the Elderly. StatPearls [Internet].

[5] Satoh M., Miura T., Shimada T., Hamazaki T. (2023). Risk Stratification for Early and Late Falls in Acute Care Settings. J. Clin. Nurs..

[6] Minsitero Della Salute (2007). Dipartimento Della Programmazione e Dell’ordinamento Del Servizio Sanitario Nazionale (SSN) Direzione Generale Della Programmazione, Ex Ufficio III. Raccomandazione per La Prevenzione e La Gestione Della Caduta Del Paziente Nelle Strutture Sanitarie, Raccomandazione n. 13, Novembre 2011 (Aggiornata Al 1 Dicembre 2011). https://www.pnes.salute.gov.it/imgs/C_17_pubblicazioni_1639_allegato.pdf.

[7] Montero-Odasso M.M., Kamkar N., Pieruccini-Faria F., Osman A., Sarquis-Adamson Y., Close J., Hogan D.B., Hunter S.W., Kenny R.A., Lipsitz L.A. (2021). Evaluation of Clinical Practice Guidelines on Fall Prevention and Management for Older Adults: A Systematic Review. JAMA Netw. Open.

[8] Ford E., Curlewis K., Squires E., Griffiths L.J., Stewart R., Jones K.H. (2021). The Potential of Research Drawing on Clinical Free Text to Bring Benefits to Patients in the United Kingdom: A Systematic Review of the Literature. Front. Digit. Health.

[9] Park Y.-J., Pillai A., Deng J., Guo E., Gupta M., Paget M., Naugler C. (2024). Assessing the Research Landscape and Clinical Utility of Large Language Models: A Scoping Review. BMC Med. Inform. Decis. Mak..

[10] Ghim J.-L., Ahn S. (2023). Transforming Clinical Trials: The Emerging Roles of Large Language Models. Transl. Clin. Pharmacol..

[11] Van Veen D., Van Uden C., Blankemeier L., Delbrouck J.-B., Aali A., Bluethgen C., Pareek A., Polacin M., Reis E.P., Seehofnerová A. (2024). Adapted Large Language Models Can Outperform Medical Experts in Clinical Text Summarization. Nat. Med..

[12] Dave T., Athaluri S.A., Singh S. (2023). ChatGPT in Medicine: An Overview of Its Applications, Advantages, Limitations, Future Prospects, and Ethical Considerations. Front. Artif. Intell..

[13] Ge W., Godeiro Coelho L.M., Donahue M.A., Rice H.J., Blacker D., Hsu J., Newhouse J.P., Hernandez-Diaz S., Haneuse S., Westover B. (2024). Automated Identification of Fall-Related Injuries in Unstructured Clinical Notes. Am. J. Epidemiol..

[14] Trinh V.Q.-N., Zhang S., Kovoor J., Gupta A., Chan W.O., Gilbert T., Bacchi S. (2023). The Use of Natural Language Processing in Detecting and Predicting Falls within the Healthcare Setting: A Systematic Review. Int. J. Qual. Health Care.

[15] Lorenzoni G., Gregori D., Bressan S., Ocagli H., Azzolina D., Da Dalt L., Berchialla P. (2024). Use of a Large Language Model to Identify and Classify Injuries With Free-Text Emergency Department Data. JAMA Netw. Open.

[16] Choi D.H., Kim Y., Choi S.W., Kim K.H., Choi Y., Do Shin S. (2024). Using Large Language Models to Extract Core Injury Information From Emergency Department Notes. J. Korean Med. Sci..

[17] Lorenzoni G., Rampazzo R., Buratin A., Berchialla P., Gregori D. (2021). Does the Integration of Pre-Coded Information with Narratives Improve in-Hospital Falls’ Surveillance?. Appl. Sci..

[18] Rudnytskyi I. (2023). Openai: R Wrapper for OpenAI API. https://irudnyts.github.io/openai/.

[19] https://Platform.Openai.Com/Docs/Guides/Prompt-Engineering/Strategy-Split-Complex-Tasks-into-Simpler-Subtasks.

[20] Denecke K., May R., Rivera Romero O., LLMHealthGroup (2024). Potential of Large Language Models in Health Care: Delphi Study. J. Med. Internet Res..

[21] Sushil M., Kennedy V.E., Mandair D., Miao B.Y., Zack T., Butte A.J. (2024). CORAL: Expert-Curated Oncology Reports to Advance Language Model Inference. NEJM AI.

[22] World Health Organization (2021). Ethics and Governance of Artificial Intelligence for Health: WHO Guidance.

[23] Topol E.J. (2019). High-Performance Medicine: The Convergence of Human and Artificial Intelligence. Nat. Med..

[24] Szolovits P. (2024). Large Language Models Seem Miraculous, but Science Abhors Miracles. NEJM AI.

[25] Chan B. (2023). Black-Box Assisted Medical Decisions: AI Power vs. Ethical Physician Care. Med. Health Care Philos..

[26] London A.J. (2019). Artificial Intelligence and Black-Box Medical Decisions: Accuracy versus Explainability. Hastings Cent. Rep..

