# Supplementary material for: A Large Language Model-Based Approach for Coding Information from Free-Text Reported in Fall Risk Surveillance Systems: New Opportunities for In-Hospital Risk Management
Source: J Clin Med. 2025 Feb 26;14(5):1580. doi: 10.3390/jcm14051580 (PMC11900557; doi:10.3390/jcm14051580)
Supplement: Supplementary file 1 [file jcm-14-01580-s001.zip › jcm-3470639-supplementary.pdf]

Supplementary Material

Table S1. Characteristics of the fall records with free-text event descriptions detailing the presence or absence of fall-related injuries. Data are absolute numbers (percentages).

**Characteristic**	**N = 93**
Witnesses of the fall	
No	44 (47%)
Yes	49 (53%)
Fall risk management plan available	
No	28 (33%)
Yes	58 (62%)
Previous in-hospital falls (same hospitalization)	
No	84 (92%)
Yes	7 (8%)
Potential causes (patient)	
Walking barefoot	17 (31%)
Open slippers	21 (39%)
Type of clothing	3 (6%)
Medical devices	3 (6%)
More than one	10 (19%)
Fall-related injury	
No	29 (31%)
Yes	64 (69%)

Table S2. Classification agreement for location. The columns report the classification according to the gold standard. The rows present the classification according to GPT for all possible combinations of the values of temperature and frequency/presence penalty. Data are absolute numbers (percentages).

**Characteristic**	**Bathroom**, N = 56	**Hospital room**, N = 126	**Hallway**, N = 5
Temperature: 0.2, Frequency/Presence penalty: 0.2			
Hallway	0 (0%)	0 (0%)	5 (100%)
Bathroom	53 (95%)	11 (8.7%)	0 (0%)
Hospital room	3 (5.4%)	115 (91%)	0 (0%)
Temperature: 0.2, Frequency/Presence penalty: 0.7			
Hallway	0 (0%)	0 (0%)	5 (100%)
Bathroom	53 (95%)	11 (8.7%)	0 (0%)
Hospital room	3 (5.4%)	115 (91%)	0 (0%)
Temperature: 0.2, Frequency/Presence penalty: 1.2			
Hallway	0 (0%)	0 (0%)	5 (100%)
Bathroom	53 (95%)	11 (8.7%)	0 (0%)
Hospital room	3 (5.4%)	115 (91%)	0 (0%)
Temperature: 0.7, Frequency/Presence penalty: 0.2			
Hallway	0 (0%)	0 (0%)	5 (100%)
Bathroom	53 (95%)	11 (8.7%)	0 (0%)
Hospital room	3 (5.4%)	115 (91%)	0 (0%)
Temperature: 0.7, Frequency/Presence penalty: 0.7			
Hallway	0 (0%)	0 (0%)	5 (100%)
Bathroom	53 (95%)	7 (5.6%)	0 (0%)
Hospital room	3 (5.4%)	119 (94%)	0 (0%)
Temperature: 0.7, Frequency/Presence penalty: 1.2			
Hallway	0 (0%)	0 (0%)	5 (100%)
Bathroom	53 (95%)	10 (7.9%)	0 (0%)
Hospital room	3 (5.4%)	116 (92%)	0 (0%)
Temperature: 1.2, Frequency/Presence penalty: 0.2			
Hallway	0 (0%)	0 (0%)	5 (100%)
Bathroom	54 (96%)	11 (8.7%)	0 (0%)
Hospital room	2 (3.6%)	115 (91%)	0 (0%)
Temperature: 1.2, Frequency/Presence penalty: 0.7			
Hallway	0 (0%)	0 (0%)	5 (100%)
Bathroom	54 (96%)	11 (8.7%)	0 (0%)
Hospital room	2 (3.6%)	115 (91%)	0 (0%)
Temperature: 1.2, Frequency/Presence penalty: 1.2			
Hallway	0 (0%)	0 (0%)	5 (100%)
Bathroom	53 (95%)	10 (7.9%)	0 (0%)
Hospital room	3 (5.4%)	116 (92%)	0 (0%)

Table S3. Classification agreement for fall-related injury. The columns report the classification according to the gold standard. The rows present the classification according to GPT for all possible combinations of the values of temperature and frequency/presence penalty. Data are absolute numbers (percentages).

	No (N=29)	Yes (N=64)
Temperature: 0.2, Frequency/Presence penalty: 0.2		
No	28 (96.6%)	2 (3.1%)
Yes	1 (3.4%)	62 (96.9%)
Temperature: 0.2, Frequency/Presence penalty: 0.7		
No	28 (96.6%)	3 (4.7%)
Yes	1 (3.4%)	61 (95.3%)
Temperature: 0.2, Frequency/Presence penalty: 1.2		
No	28 (96.6%)	2 (3.1%)
Yes	1 (3.4%)	62 (96.9%)
Temperature: 0.7, Frequency/Presence penalty: 0.2		
No	28 (96.6%)	2 (3.1%)
Yes	1 (3.4%)	62 (96.9%)
Temperature: 0.7, Frequency/Presence penalty: 0.7		
No	28 (96.6%)	2 (3.1%)
Yes	1 (3.4%)	62 (96.9%)
Temperature: 0.7, Frequency/Presence penalty: 1.2		
No	28 (96.6%)	1 (1.6%)
Yes	1 (3.4%)	63 (98.4%)
Temperature: 1.2, Frequency/Presence penalty: 0.2		
No	28 (96.6%)	2 (3.1%)
Yes	1 (3.4%)	62 (96.9%)
Temperature: 1.2, Frequency/Presence penalty: 0.7		
No	28 (96.6%)	2 (3.1%)
Yes	1 (3.4%)	62 (96.9%)
Temperature: 1.2, Frequency/Presence penalty: 1.2		
No	29 (100.0%)	2 (3.1%)
Yes	0 (0.0%)	62 (96.9%)

Table S4. Example of record misclassified (fall in the hospital bedroom misclassified as fall in the bathroom)

Text (English)
The patient (with his wife present) slipped and fell backwards while going to the bathroom and hit his head. Called by his wife, we found him on the ground near the bed, awake and cooperative. We put him in bed with an ice pack.
